# Sirt1/Foxo Axis Plays a Crucial Role in the Mechanisms of Therapeutic Effects of Erzhi Pill in Ovariectomized Rats

**DOI:** 10.1155/2018/9210490

**Published:** 2018-08-26

**Authors:** Wenna Liang, Xihai Li, Guanhui Li, Liu Hu, Shanshan Ding, Jie Kang, Jianying Shen, Candong Li, Tetsuya Asakawa

**Affiliations:** ^1^Research Base of Traditional Chinese Medicine Syndrome, Fujian University of Traditional Chinese Medicine, Fuzhou 350122, China; ^2^Academy of Integrative Medicine, Fujian University of Traditional Chinese Medicine, Fuzhou, Fujian 350122, China; ^3^Fujian Key Laboratory of Integrative Medicine on Geriatrics, Fujian University of Traditional Chinese Medicine, Fuzhou 350122, China; ^4^Department of Neurosurgery, Hamamatsu University School of Medicine, Handayama, 1-20-1, Higashi-ku, Hamamatsu, Shizuoka 431-3192, Japan

## Abstract

*Background*. Erzhi pill (EZP), a traditional Chinese herbal formula, has been widely used to treat postmenopausal osteoporosis (PMOP) in China. However, its molecular mechanisms remain unclear. The aim of the present study is to investigate the antiosteoporotic effect of EZP on an ovariectomized rat model of PMOP. We performed the biomarkers of bone metabolism disorder, bone morphology, bone mineral density (BMD), and bone biomechanics to confirm the successful establishment of the PMOP model. We then investigated the expression of biomarkers related to the Sirt1/Foxo axis. We also examined microRNA-132 (miR-132), a regulator in the Sirtuin1 (Sirt1) expression. The bone metabolism disorder, bone morphology, BMD, and bone biomechanics in ovariectomized rats were improved by EZP administration. The antiosteoporotic effect of EZP was confirmed. We also found that the expressions of Sirt1, Runx2, Foxo1, and Foxo3a were downregulated in ovariectomized rats, while being then upregulated by EZP administration. And the expression of PPAR-*γ* and miR-132 was upregulated in ovariectomized rats and then downregulated by EZP administration. These results provided evidence that Sirt1/Foxo axis related mechanism may play a crucial role in the therapeutic effects of EZP, indicating that Sirt1/Foxo axis can be considered as a potential therapeutic target for PMOP in the future.

## 1. Introduction

Postmenopausal osteoporosis (PMOP) as a severe public health problem is a progressive systemic skeletal disease characterized by low bone mass and microarchitectural deterioration of bone tissue quite common in postmenopausal women [1–3]. The increased bone fragility and susceptibility to fracture as clinical manifestations are caused by the imbalance of osteoblastic formation and osteoclastic resorption as a result of decreased estrogen level and ovarian function [4]. Therefore, it has been urgent to develop the availability of effective and safe treatments for PMOP. In this context, some Chinese herbs containing multiple bioactive components to antiosteoporosis were taken into account especially in China.

Antiosteoporosis has been focused on osteoblast activity and bone resorption processes, followed by osteogenesis of bone marrow mesenchymal stem cells (BMSCs) [5, 6]. BMSCs derived from PMOP exhibit impaired differentiation ability and disturbed intrinsic properties [7], which can be triggered by the Sirtuin1 (Sirt1)/Foxo axis. The relationship between osteogenesis and Sirt1/Foxo axis has been well documented. Resveratrol promotes osteogenesis of human mesenchymal stem cells by upregulating Runx2 gene expression by the Sirt1/Foxo axis [8, 9], indicating that promotion of the Sirt1/Foxo axis might play a role in the mechanisms of some Chinese herbs.

Erzhi pill (EZP) is a Chinese herbal compound formula composed of Ligustrum (*the fruit of Ligustrum lucidum Ait.*) and Yerbadetajo Herb (*the herb of Eclipta prostrata L.*). In traditional Chinese medicine (TCM), it has been widely used for antiosteoporosis in China [10]. However, the underlying mechanisms by which EZP inhibits the bone loss remain to be elucidated, and this has limited its wider use. In the present study, we therefore investigate the antiosteoporotic effects of EZP in ovariectomized rats, a PMOP model. We attempt to observe whether the EZP can inhibit the bone metabolism disorder and improve the bone mass. Moreover, we want to know the effects of EZP on the biomarkers of the Sirt1/Foxo axis, which may clarify the underlying therapeutic mechanisms of EZP.

## 2. Materials and Methods

### 2.1. Ethics Statement

All rats were treated as per the National Institute of Health Guideline for the Care and Use of Laboratory Animals. All experiments were approved by the Animal Care and Use Committee of the Fujian University of TCM. All surgical procedures were performed under 10% chloral hydrate anesthesia, and all efforts were made to minimize suffering.

### 2.2. Preparation of EZP

EZP composed of Ligustrum and Yerbadetajo Herb, obtained from the Third People's Hospital of Fujian University of TCM (Fuzhou, China), was dried in an air-circulating oven (model SFG-02.600; Hengfeng Medical Instrument Co., Ltd., Huangshi, China) at 50°C for 24 h and then crushed to an appropriate particle size with a 40 mesh screen in a high-speed rotary cutting mill (model ZN-400A; Zhongnan Pharmaceutical Machinery Factory, Changsha, China). Equal proportions of Ligustrum and Yerbadetajo Herb were added 10 times the amount of 65% ethanol for 1 h and extracted 3 times and 10 times the amount of distilled water by refluxing twice, for 2 h each time. The filtrate was evaporated on a rotary evaporator (model RE-2000; Shanghai Yarong Biochemistry Instrument Factory, Shanghai, China) and then concentrated into it per milliliter, which is equivalent to 1g of raw Chinese herbs. The working concentrations of EZP (0.3 g/ml) were made by diluting the concentrated solution in normal saline and storing at 4°C.

**Figure 1:**
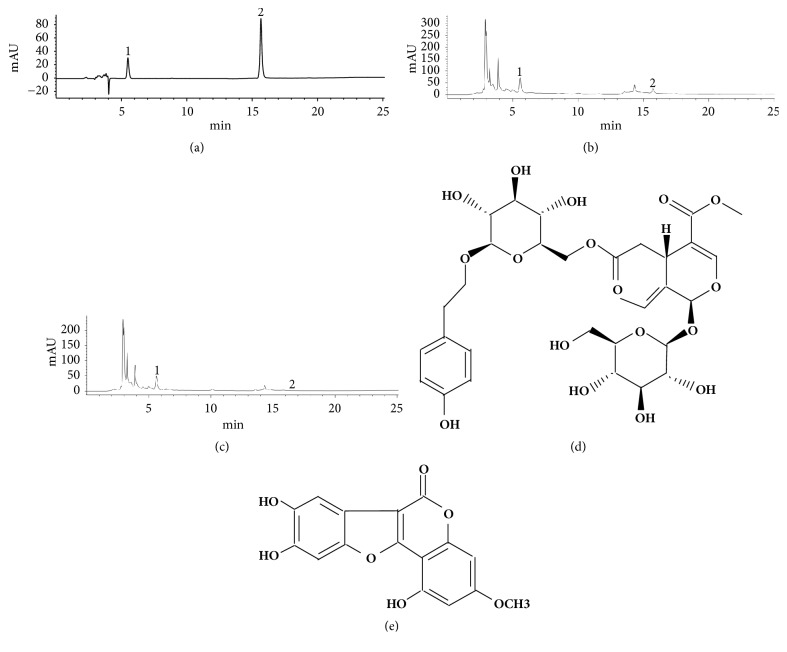
High performance liquid chromatogram of EZP. (a) The liquid chromatogram of the reference substance was composed of 2 peaks, i.e., specnuezhenide (peak 1) and wedelolactone (peak 2). (b) The 2 peaks were also found in the liquid chromatogram of EZP ethanol extract. (c) The 2 peaks were also found in the liquid chromatogram of EZP water extract, and the results showed that the content of specnuezhenide and wedelolactone in the EZP ethanol extract was higher than those in the EZP water extract. (d) The structural formula of specnuezhenide. (e) The structural formula of wedelolactone.

The quality control of the EZP extracts was analyzed by high performance liquid chromatography (HPLC) fingerprint on an Agilent 1200 HPLC system (Agilent, Santa Clara, CA, USA) using an Agilent HC-C18 column (4.60x250.00 mm, 5 *μ*m) (Figures 1(a)–1(c)). The conditions for analysis were methanol-0.1% phosphoric acid as a mobile phase and a detection wavelength at 230 nm for specnuezhenide (purity 98%, Figure 1(d)) and wedelolactone (purity 98%, Figure 1(e)) (National Institute for Pharmaceutical and Biological Products Control, Beijing, China), a flow rate of 1 ml/min, and a column temperature of 30°C.

### 2.3. Animals and Administration

60 female Sprague Dawley rats (certification no. SCXK: 2012-0002) (SPF, Shanghai Slack Laboratory Animal Co., Ltd., Shanghai, China) were housed in humidity- (55%) and temperature-controlled (23°C) rooms with a 12 h light/dark cycle and food and water supply. All rats were randomly divided into three groups, consisting of 20 rats per group, as follows: sham, ovariectomized (OVX), and OVX + EZP groups. Bilateral ovariectomy was performed in 40 female rats through an incision in the back, under general anesthesia with an intraperitoneal injection of 10% chloral hydrate at a dose of 3 ml/kg (Chemical Reagent Co., Shanghai, China). Approximately 1.5 cm of the skin, the abdominal cavity, and the muscles were incised, and the ovaries were exposed. The oviduct was ligated with a silk thread and the ovariectomy was performed bilaterally, while the remaining 20 animals underwent a sham surgery in which the bilateral ovaries were examined and returned to the original position under the same protocol.

After 2 weeks, according to the Human and Rat Equivalent Dose Conversion Principle, rats belonging to OVX + EZP group were administered 0.3 g/ml EZP (3 ml/kg) orally by gavage, whereas those belonging to the sham and OVX groups were given saline (3 ml/kg) orally by gavage. The rats underwent treatment for 12 weeks.

### 2.4. Tissue Preparation

All rats were deeply anesthetized with an intraperitoneal injection of 10% chloral hydrate (3 ml/kg). Blood was drawn from the inferior vena cava, added to anticoagulant, and centrifuged at 1,500 g for 20 min at 4°C to extract the blood serum. The extracted blood serum was stored at -70°C until analysis.

The fifth lumbar vertebra (L5) was collected and fixed in 4% paraformaldehyde (PFA)/phosphate-buffered saline (PBS) for hematoxylin-eosin (HE) staining and micro-computed tomography (micro-CT) analysis. The femur and tibia were collected and stored in ice-cold PBS for biomechanical analysis. The lumbar vertebra was removed, immediately frozen in liquid nitrogen, and stored at -70°C, for mRNA and protein detection.

### 2.5. Micro-CT and HE Staining

The microarchitecture of trabecular bone in the L5 was analyzed by micro-CT (60 KV, 50 W; ZKKS-MCT-Sharp-I, Guangzhou Zhongke Kaisheng Medical Technology Co., Ltd.). The same specimen was scanned to obtain different section images and the L5 scans were performed in three spatial dimensions. Micro View software (3-DMed 4.1) was used to calculate the following parameters: BMD, bone volume/total volume (BV/TV), trabecular thickness (Tb.Th), and trabecular separation (Tb.Sp).

The L5 was fixed and decalcified with 10% ethylene diamine tetraacetic acid (EDTA) (Sinopharm Chemical Reagent Co., Ltd., Shanghai, China), then dehydrated through a graded ethanol series, cleared with xylene, embedded in paraffin, and coronally sectioned at a thickness of 10 *μ*m. For histological analysis of L5, serial sections were subjected to standard HE staining.

### 2.6. Three-Point Bending Test

The biomechanical properties of tibia and femur were determined in a three-point bending test by a universal testing machine (Zwick/Roell Z020, Germany). Briefly, the load was applied to the femur and tibia with a load span of 20 mm until the bone fractured at a crosshead speed of 1 mm/min. The maximum load and fracture load were recorded as the maximum tibia and femur withstand load and fracture load.

### 2.7. Enzyme Linked Immunosorbent Assay (ELISA) Analysis

Serum estradiol (E2), sex hormone-binding globulin (SHBG), receptor activator of NFKB ligand (RANKL), osteoprotegerin (OPG), and bone alkaline phosphatase (BALP) levels were performed using an ELISA kit (Tsz Biosciences, Inc., Boston, USA) according to the manufacturer's instructions. The samples in 100 *μ*l were used for measurement with a microplate spectrophotometer (Omega Bio-Tek, Inc., Norcross, GA, USA) at 405 nm.

### 2.8. Expressions of Biomarker Related to the Sirt1/Foxo Axis

Real-time polymerase chain reaction (PCR) assay and western blot assay were used to measure the expressions of Sirt1/Foxo axis related biomarkers (mRNA and protein), including Sirt1, Runx2, Foxo1, Foxo3a, and PPAR-*γ*.

Total RNA was extracted from the lumbar vertebra with the liquid nitrogen grinding method using the TRIzol reagent (Invitrogen, USA). We used 1 *μ*g RNA to transcribe into cDNA using the reverse transcription kit (Thermo Fisher Scientific, Inc., USA.), and the cDNA was used for real-time PCR to determine the mRNA expression with the SYBR Florescence Quantization Kit (Invitrogen, USA) by a 7500 RT-qPCR System (Applied Biosystems, USA). The primers were listed as follows: Sirt1 forward, 5′- AGG AAG TCA GCA TCG CAT TG -3′ and reverse, 5′-AGG CTC TAC CAC AGT GAT AGG-3′; Runx2 forward, 5′-TCC AGC CAC CTT CAC TTA CA-3′ and reverse, 5′-TCA GCG TCA ACA CCA TCA TTC-3′; Foxo1 forward: 5′-AAT TTG CTA AGA GCC GAG GA-3′ and reverse: 5′-AAG TCA TCA TTG CTG TGG GA-3; Foxo3a 5′-TAC GAG TGG ATG GTG CGC TG-3′ and 5′-AGG TTG TGG CGG ATG GAG TTC-3′; PPAR-*γ* forward, 5′-CTG CTC CAC ACT ATG AAG ACA TC-3′ and reverse, 5′-TCC GAC AGT TAA GAT CAC ACC TAT -3′ GAPDH forward, 5′-ACG GCA AGT TCA ACG GCA CAG-3′ and reverse, 5′- GAA GAC GCC AGT AGA CTC CAC GAC-3′. Fold changes were calculated by the formula 2^−ΔΔCt^ relative to the expression in sham group and GAPDH was the endogenous control.

Total protein was extracted from the lumbar vertebra with the liquid nitrogen grinding method using the radioimmunoprecipitation assay (RIPA) lysis buffer (Beyotime Biotech, China) with phenylmethanesulfonyl fluoride (PMSF; Beyotime Biotech, China) 1 mM, and the protein concentrations were measured using the bicinchoninic acid assay (BCA). An equal amount of protein was separated by electrophoresis on 12% sodium dodecyl sulfate-polyacrylamide gel electrophoresis (SDS-PAGE) (Sigma-Aldrich, USA) and electrotransferred onto PVDF membranes (Thermo Fisher Scientific, Inc., USA). The membranes were blocked with 5% nonfat milk; incubated with the primary antibodies against Sirt1 (9475), Runx2 (12556), Foxo1 (2880), Foxo3a (12839), and PPAR-*γ* (2435, including PPAR-*γ*1 and PPAR-*γ*2) (all from Cell Signaling Technology, Inc., Beverly, MA, USA) and *β*-actin (sc 47778; Santa Cruz Biotechnology, Inc., USA) at 4°C overnight; and then incubated with the horseradish peroxidase- (HRP-) conjugated secondary antibody IgG (ZB-2301; Zhongshan Goldenbridge Biotech, China) at room temperature. Data were quantitated by a Bio-Rad ChemiDoc XRS+ (Bio-Rad, USA) and then compared with *β*-actin as normalization.

### 2.9. Real-Time PCR Assay

Expression of miR-132 was measure using a TaqMan microRNA assay, a real-time PCR. Total RNA was extracted as per the instruction of the mirVana™ isolation kit (Invitrogen, USA). Reverse transcription was performed with miRNA specific stem loop RT primers and the TaqMan microRNA reverse transcription kit (Applied Biosystems, USA). The miR-132 expression was detected using the TaqMan Universal PCR Master (Applied Biosystems, USA). Fold changes of target miRNA were calculated relative to the expressions in those of untreated cells, and U6 was used as the endogenous control.

### 2.10. Statistical Analysis

SPSS software (version 17.0; SPSS Inc., USA) was employed to perform the statistical analysis. One-way analysis of variance and the Student-Newman-Keuls q test were used for the multiple comparisons. All data were presented as the mean ± standard deviation (SD);* P*<0.05 was considered as a statistically significant difference.

## 3. Results

### 3.1. Conformation of the Therapeutic Effects of EZP on the Ovariectomized Rat Model

#### 3.1.1. EZP Inhibited the Body Weight Gain in the Ovariectomized Rat Model

**Figure 2:**
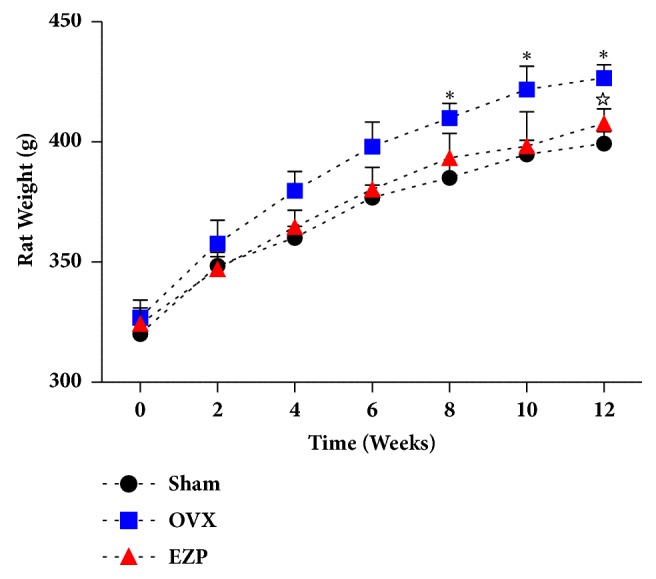
EZP inhibited the body weight gain in the ovariectomized rat model. Rat weight of OVX group was higher than that of sham group from 8 week. Rat weight of EZP group was lower than that of OVX group at 12 week. Body weight was expressed as mean** ±** SD. *∗*P<0.05, versus sham group; ^☆^P<0.05, versus OVX group.

Body weights of rats were monitored every two weeks during the administration of EZP in 12 weeks (Figure 2); the increase of body weight in OVX group was significantly higher than that of the sham group, and that of the EZP group was significantly lower than that of OVX group.

#### 3.1.2. EZP Improved the Estrogen Deficiency Mediated Bone Metabolism Disorder in the Ovariectomized Rat Model

**Figure 3:**
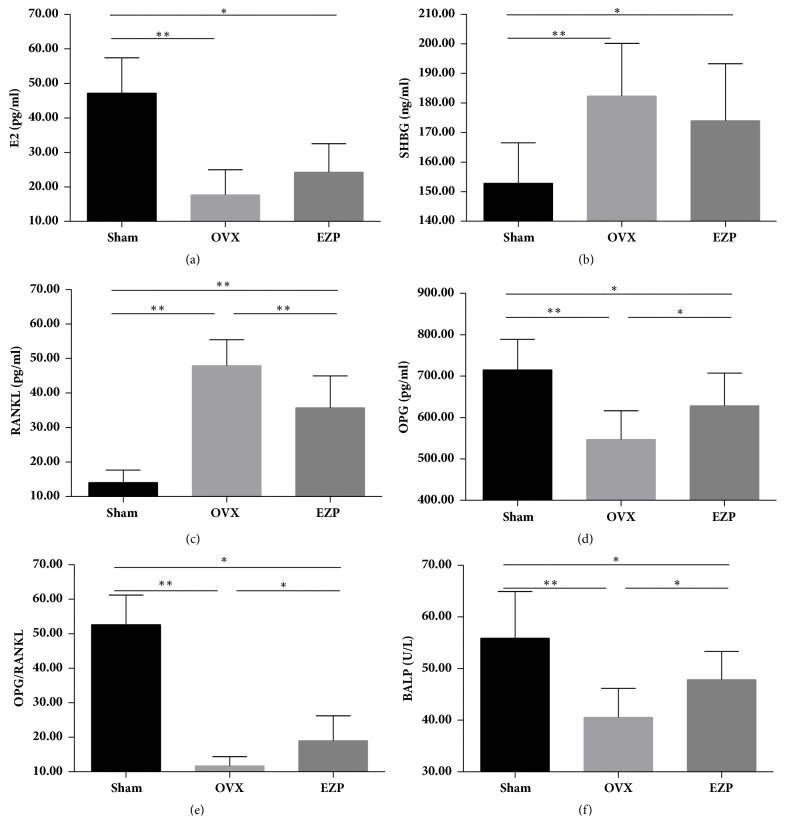
EZP improved the estrogen deficiency mediated bone metabolism disorder in the ovariectomized rat model. Results of ELISA assay show the serum concentration of E2 (a), SHBG (b), RANKL (c), OPG (d), OPG/RANKL (e), and BALP (f). EZP downregulated serum concentration of RANKL and upregulated serum concentration of OPG, OPG/RANKL, and BALP. *∗* means P<0.05; *∗∗* means P<0.01.

The serum E2 level of OVX group was lower than that of sham group, and the serum SHBG level displayed the contrary tendency (Figures 3(a) and 3(b)); the serum OPG, OPG/RANKL, and BALP of OVX group was lower than those of sham group, and the serum RANKL showed the contrary tendency (Figures 3(c)–3(f)), which provided other evidence of our successful establishment of the estrogen deficiency mediated bone metabolism disorder in the ovariectomized rat model.

Interestingly, there were no differences in the serum E2 and SHBG levels after administration of EZP. However, after administration of EZP, the biomarkers of bone metabolism RANKL in serum was were lower than those of OVX group, and the OPG, OPG/RANKL, and BALP in serum were higher than those of OVX group.

#### 3.1.3. EZP Enhanced the Biomechanical Function of Tibia and Femur in the Ovariectomized Rat Model

Tibia break point and femur break point of the OVX group were lower than that of sham group, which provided evidence of our successful establishment of the ovariectomized rat model.

**Figure 4:**
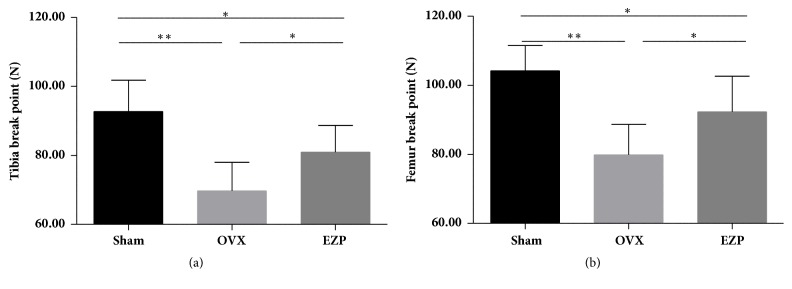
EZP enhanced the tibia break point and femur break point. The tibia break point (a) and femur break point (b) were measured by the three-point bending test. *∗* means P<0.05; *∗∗* means P<0.01.

After administration of EZP, upregulated tibia break point and femur break point were compared in the OVX group (Figure 4).

#### 3.1.4. EZP Reduced the Bone Loss in the Ovariectomized Rat Model

**Figure 5:**
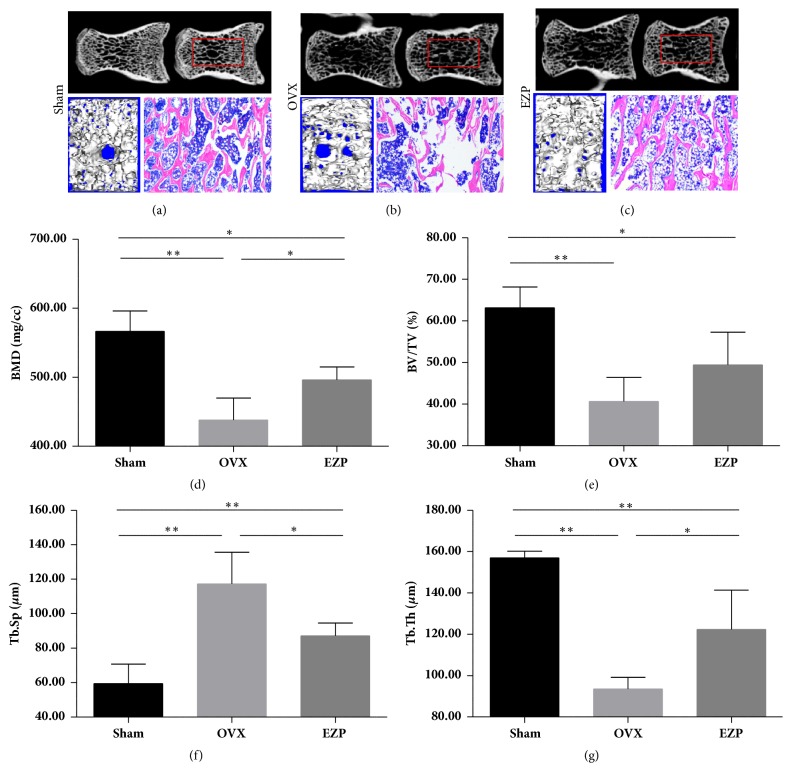
EZP improves trabeculae of L5. Microarchitecture of L5 was analyzed using micro-computed tomography and HE staining (a–c). EZP upregulated the BMD (d) and Tb.Th (f) and downregulated the Tb.Sp (g). There was no BV/TV difference in EZP group and OVX group (e). *∗* means P<0.05; *∗∗* means P<0.01.

The images with micro-CT and results from HE staining of the L5 of the OVX group revealed a significantly decreased trabecular bone volume compared with the sham group (Figures 5(a)–5(c)). The BMD, BV/TV, and Tb.Th of OVX group were lower than those of sham group (Figures 5(d)–5(f)), and the Tb.Sp displayed the contrary tendency (Figure 5(g)), which provided evidence of our successful establishment of the ovariectomized rat model.

After administration of EZP, the BMD and Tb.Th were upregulated and the Tb.Sp was downregulated compared with the OVX group. Moreover, HE staining of the L5 of the OVX group exhibited significantly reduced furcation and a relatively scant marrow space compared with those in the sham group; this result is consistent with the micro-CT data. 

### 3.2. Evidence of the Mechanisms May Be Associated with the Sirt1/Foxo Axis

#### 3.2.1. EZP Changed the Expressions of Biomarkers Related to the Sirt1/Foxo Axis

**Figure 6:**
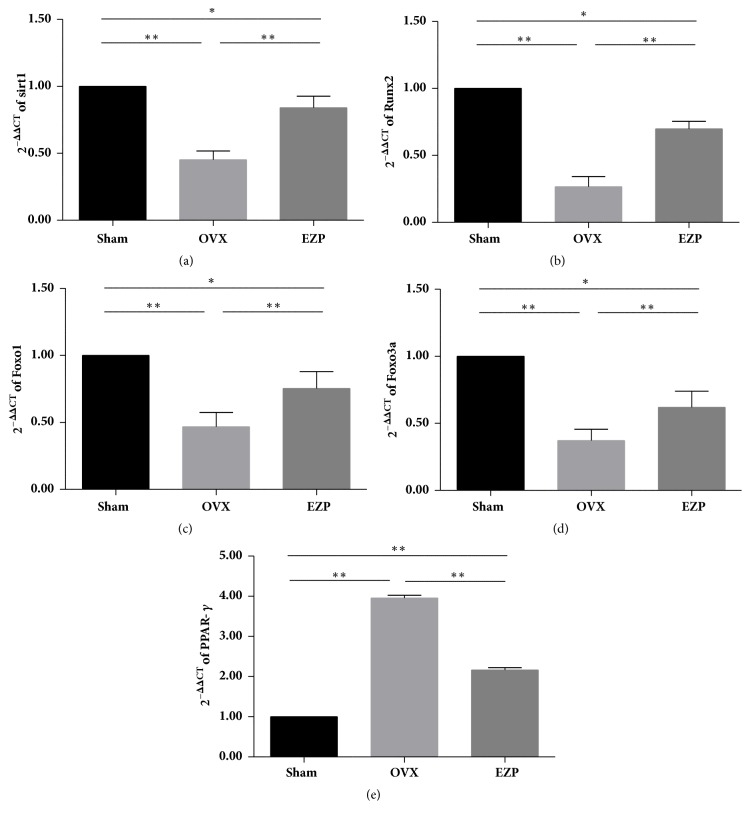
EZP upregulated mRNA expressions of Sirt1, Runx2, Foxo1, and Foxo3a and downregulated mRNA expression of PPAR-*γ*. Results of real-time PCR show the mRNA expressions of Sirt1 (a), Runx2 (b), Foxo1 (c), Foxo3a (d), and PPAR-*γ* (e) affected by EZP administration. GAPDH was used as the internal control for the quantification analysis. Data are the means ± standard deviation (SD) and SD is shown as vertical bars. *∗* means P<0.05; *∗∗* means P<0.01.

**Figure 7:**
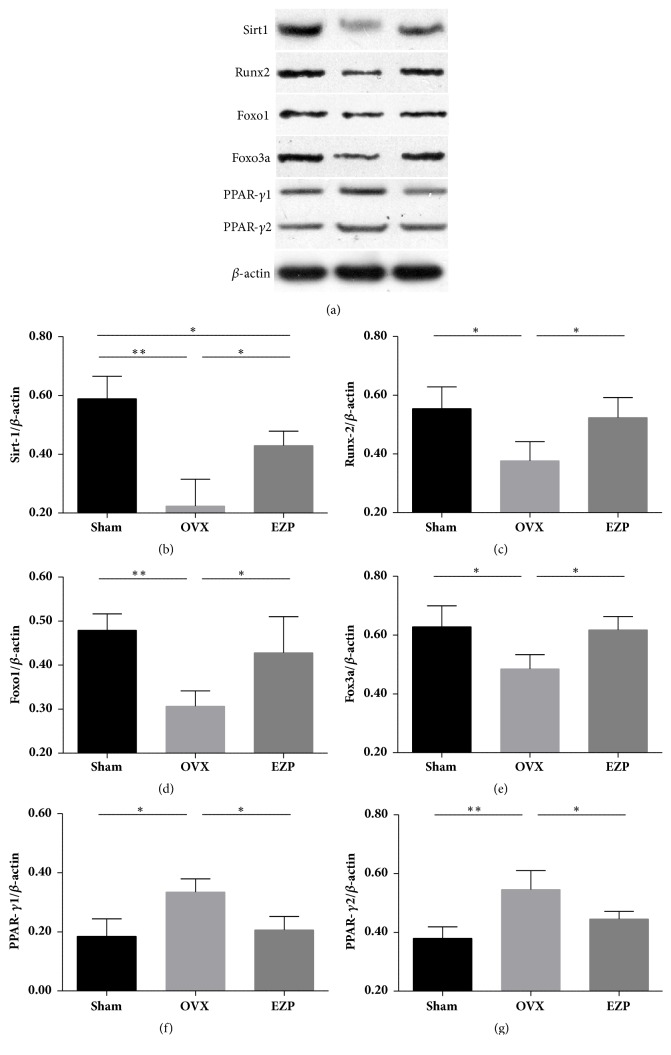
EZP upregulated protein levels of Sirt1, Runx2, Foxo1, and Foxo3a and downregulated protein level of PPAR-*γ*. Results of western blot analysis show the expressions of proteins of Sirt1, Runx2, Foxo1, Foxo3a, and PPAR-*γ* affected by EZP administration (a–g). *β*-actin was used as the internal control for the quantification analysis. Data are the means ± standard deviation (SD) and SD is shown as vertical bars. *∗* means P<0.05; *∗∗* means P<0.01.

Both the results of mRNA (Figure 6) and protein (Figure 7) exhibited the same tendency. All the key regulators of the Sirt1/Foxo axis exhibited significant difference with the sham group, which indicated the successful establishment of the ovariectomized rat model.

The expressions of both mRNA and protein of Sirt1, Runx2, Foxo1, and Foxo3a were upregulated by administration of EZP (Figures 6(a)–6(d) and 7(b)–7(e)). The expressions of both mRNA and protein of PPAR-*γ* were downregulated by administration of EZP (Figures 6(e) and 7(f) & 7(g)). These changes are associated with the changes of Sirt1/Foxo axis, which is therefore speculated to play a role in the mechanisms of effects of EZP.

#### 3.2.2. EZP Downregulated Expressions of miR-132 of the Ovariectomized Rat Model

**Figure 8:**
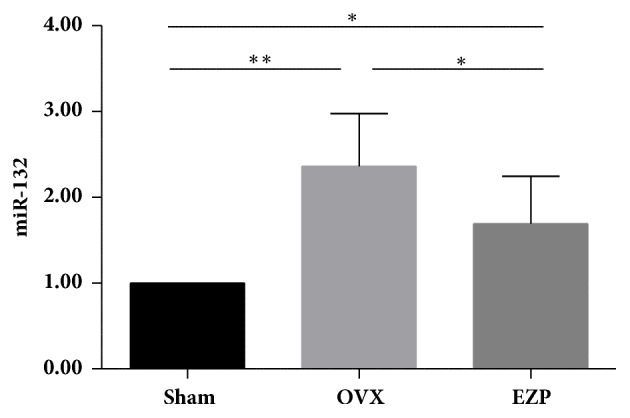
EZP downregulated expression of miR-132 in an ovariectomized rat model. Expression of miR-132 was measured by a real-time PCR. Data are the means ± standard deviation (SD) and SD is shown as vertical bars. *∗* means P<0.05; *∗∗* means P<0.01.

The results of TaqMan microRNA assay showed that expressions of the miR-132 in the OVX group were significant higher than those of the sham group (Figure 8), which indicated the successful establishment of the ovariectomized rat model.

We found that administration of EZP significantly downregulated the expression of miR-132 in the ovariectomized rat model. This result may provide evidence that Sirt1/Foxo axis plays a role in the mechanisms of effects of EZP by regulating the expression of miR-132.

## 4. Discussion

Using an ovariectomized rat model as PMOP model, there were two findings in the present study: The first one was that we confirmed the antiosteoporotic effects of EZP on the ovariectomized rat model, and such effects include four aspects, namely, inhibiting the body weight gain, improving the bone metabolism disorder, enhancing the biomechanical function, and reducing the bone loss. The second finding was that we confirmed that the Sirt1/Foxo axis may play a role in the mechanisms of EZP production, since we found the expressions of PPAR-*γ* and miR-132 downregulated, whereas the expressions of Sirt1, Runx2, Foxo1, and Foxo3a upregulated by EZP administration. The present study provided evidence of the antiosteoporotic effects of EZP. Moreover, the Sirt1/Foxo axis related mechanisms may uncover the tip of the iceberg of the complex mechanisms underlying the therapeutic effect of a battery of Chinese herbs with “nourishing liver and kidney” represented by EZP. To the best of our knowledge, this is the first report confirming the antiosteoporotic effects and Sirt1/Foxo axis related mechanism on EZP. The findings of this study may contribute to further investigation of EZP, which is commonly used in the clinical practice against PMOP.

It has been well documented that the estrogen deficiency mediated bone metabolism disorder is closely associated with the onset and progression of PMOP [11, 12]. Treatments promoting the bone formation or inhibiting the bone resorption can achieve amelioration of PMOP, while estrogen deficiency may induce deterioration of PMOP. The ovariectomized rat model can simulate the pathological process of bone loss during the postmenopause stage in humans, and it has been widely used to evaluate the effects of drugs in PMOP [13, 14]. We employed bilateral ovariectomy to establish the PMOP animal model, and the results are in accordance with these previous conclusions. After 12 weeks, our data exhibited that the E2, OPG, OPG/RANKL, BALP, BMD, and Tb.Th were significantly reduced, and the SHBG, RANKL, and Tb.Sp were significantly increased, which confirmed the successful establishment of the PMOP animal model. Then, EZP was administrated for confirmation of the efficacy. We found that EZP can improve OPG, OPG/RANKL, BALP, BMD, and Tb.Th which was suppressed by estrogen deficiency, and the biomechanical function reduction was also suppressed. The antiosteoporotic effect of EZP is therefore verified. Thus, we successfully established a model to reproduce the clinical efficacy of EZP in* vitro*.

A variety of physiological and pathological signals could affect the function of osteoblast and osteoclast, which can then influence the function and structure of the bone [15, 16]. A precise balance between bone formation and resorption is required for bone remodeling. RANKL can induce the differentiation of progenitor cells into mature osteoclast [17, 18]. Conversely, OPG can inhibit the binding of a RANKL to the RANK receptor [19]. Therefore, RANKL/RANK/OPG pathway has been an important treatment way for PMOP [20, 21]. The balance between bone formation and resorption is maintained by regulating the ratio of OPG/RANKL [22]. Our data showed that the decrease of the ratio of OPG/RANKL was improved by the administration of EZP.

Many signal pathways are involved in the mechanisms of bone metabolism disorder. Sirt1/Foxo axis regulates the BMSCs change to osteogenesis rather than adipogenesis [8]. Sirt1, a member of the sirtuin family of Nicotinamide adenine dinucleotide (NAD+)-dependent deacetylases, is found to be an antiaging gene, which is one of the novel molecular targets [23]. Sirt1 deacetylates various transcription factors in the nucleus to control metabolism, cell survival, and cell differentiation [24–26]. Foxo3a, a member of the multifunctional Foxo family of transcription factors, is an important target of Sirt1 [27, 28]. The transcription factors PPAR-*γ*2 and Runx2 are regarded as critical in initiating BMSCs change to osteogenesis and adipogenesis [29, 30]. PPAR-*γ*2 has been shown to play a key role in inhibiting osteoblast differentiation by multitudinous gene-gene interactions [31]. Sirt1 enhances osteogenic differentiation indirectly by inhibiting adipocyte formation and repressing PPAR-*γ*2 expression [32, 33]. PPAR-*γ*2 also interferes with Runx2-mediated transactivation activity and represses Runx2 mRNA transcription in osteoblast [9, 34], and then a regulatory effect of Sirt1 on Runx2 transcription also exists during the resveratrol-mediated osteogenesis in BMSCs. Our results on EZP are highly in accordance with this study. The expression of PPAR-*γ* was downregulated, while the expressions of Sirt1, Runx2, Foxo1, and Foxo3a were upregulated by administration of EZP.

Moreover, we found that expression of miR-132 was also upregulated. The network and interaction of Sirt1/Foxo axis are quite complicated in bone formation and resorption. miR-132 plays a crucial role in regulating osteogenic differentiation by a target gene of Sirt1[35, 36]. Overexpression of miR-132 also suppressed ALP activity, and Col1a1 and Runx2 expression; reciprocally, inhibition of miR-132 contributed to the recovery of osteogenic differentiation [37]. Using microarray profiling, it has been found that miR-132 was obviously upregulated in primary rat osteoblasts cultured in a simulated microgravity environment, which could suppress the osteoblast differentiation. The negative effect of simulated microgravity on osteoblast differentiation was blocked by the inhibition of miR-132 [38]. Summarizing the results of change of the miR-132, we can get a strong evidence that regulation of the Sirt1/Foxo axis is an important mechanism involved in the therapeutic effects of EZP on the PMOP.

The pathophysiological changes of PMOP are quite complicated. We believe that some of the mechanisms of bone formation, but not all, are crucial. Furthermore, not only the Sirt1/Foxo axis, but many other signaling pathways are also involved in the PMOP pathogenesis. The interaction of such pathways must be complicated and individual. In this regard, we believe that the mechanisms of EZP are also very complex and multifold. The findings of the present study just uncover a tip of the iceberg. To clarify the detailed mechanisms of the therapeutic effects of EZP, more signaling pathways in PMOP models should be fully verified, which will be included in our future works.

There is a potential limitation of this study. We had never checked the safety of treatment concentration of EZP in animals. Indeed, EZP is a Chinese herbal compound formula, which has been widely used for antiosteoporosis in China. In this study, we used the clinical equivalent dose of EZP according to the Human and Rat Equivalent Dose Conversion Principle. Since no changes in renal and liver function of EZP were reported in human being, we therefore omitted examinations of them in animals. This might be a potential limitation in the present study. We will check renal and liver function in animals during the drug treatment in our future experiments.

## 5. Conclusion

The present study confirmed the therapeutic effects of EZP on a ovariectomized rat model. We also proved that Sirt1/Foxo axis related mechanism may play a role in the antiosteoporotic effect of EZP. EZP is a representative of a group of Chinese herbs with the functions of “nourishing liver and kidney”. The present study provided a useful animal class model to verify the efficacy and mechanisms for these herbs. We expected that rigorously designed studies concerning these herbs in the levels of human patients will be performed in the future, which may provide powerful evidence and uncover the mechanisms for such Chinese herbs, since they are commonly used in China to treat PMOP.

## Data Availability

The data used to support the findings of this study are available from the corresponding author upon request.
